# Dimethyl Fumarate, an Approved Multiple Sclerosis Treatment, Reduces Brain Oxidative Stress in SIV-Infected Rhesus Macaques: Potential Therapeutic Repurposing for HIV Neuroprotection

**DOI:** 10.3390/antiox10030416

**Published:** 2021-03-09

**Authors:** Yoelvis Garcia-Mesa, He N. Xu, Patricia Vance, Analise L. Gruenewald, Rolando Garza, Cecily Midkiff, Xavier Alvarez-Hernandez, David J. Irwin, Alexander J. Gill, Dennis L. Kolson

**Affiliations:** 1 Department of Neurology, Perelman School of Medicine, University of Pennsylvania, Philadelphia, PA 19104, USA; megarcia@pennmedicine.upenn.edu (Y.G.-M.); vancep2@patriciavance.com (P.V.); analise.gruenewald@gmail.com (A.L.G.); garzar18@livemail.uthscsa.edu (R.G.); dirwin@pennmedicine.upenn.edu (D.J.I.); agill@pennmedicine.upenn.edu (A.J.G.); 2 Britton Chance Laboratory of Redox Imaging, Department of Radiology, Perelman School of Medicine, University of Pennsylvania, Philadelphia, PA 19104, USA; hexu2@pennmedicine.upenn.edu; 3 Department of Pathology, TNPRC, Tulane University, Covington, LA 70433, USA; cconerly@tulane.edu (C.M.); jalvarezhernandez@txbiomed.org (X.A.-H.)

**Keywords:** SIV, HIV-associated neurocognitive disorders, fumaric acid esters, dimethyl fumarate, DMF, oxidative stress, antioxidant response, optical redox ratio, macaque, brain

## Abstract

Dimethyl fumarate (DMF), an antioxidant/anti-inflammatory drug approved for the treatment of multiple sclerosis, induces antioxidant enzymes, in part through transcriptional upregulation. We hypothesized that DMF administration to simian immunodeficiency virus (SIV)-infected rhesus macaques would induce antioxidant enzyme expression and reduce oxidative injury and inflammation throughout the brain. Nine SIV-infected, CD8^+^-T-lymphocyte-depleted rhesus macaques were studied. Five received oral DMF prior to the SIV infection and through to the necropsy day. Protein expression was analyzed in 11 brain regions, as well as the thymus, liver, and spleen, using Western blot and immunohistochemistry for antioxidant, inflammatory, and neuronal proteins. Additionally, oxidative stress was determined in brain sections using immunohistochemistry (8-OHdG, 3NT) and optical redox imaging of oxidized flavoproteins containing flavin adenine dinucleotide (Fp) and reduced nicotinamide adenine dinucleotide (NADH). The DMF treatment was associated with no changes in virus replication; higher expressions of the antioxidant enzymes NQO1, GPX1, and HO-1 in the brain and PRDX1 and HO-2 in the spleen; lower levels of 8-OHdG and 3NT; a lower optical redox ratio. The DMF treatment was also associated with increased expressions of cell-adhesion molecules (VCAM-1, ICAM-1) and no changes in HLA-DR, CD68, GFAP, NFL, or synaptic proteins. The concordantly increased brain antioxidant enzyme expressions and reduced oxidative stress in DMF-treated SIV-infected macaques suggest that DMF could limit oxidative stress throughout the brain through effective induction of the endogenous antioxidant response. We propose that DMF could potentially induce neuroprotective brain responses in persons living with HIV.

## 1. Introduction

Despite the effectiveness of combination antiretroviral therapy (cART) in suppressing human immunodeficiency virus (HIV) replication in the peripheral and central nervous system (CNS) compartments, individuals remain at risk for secondary complications that are related to persistent or recurrent inflammation and oxidative stress in all compartments [1,2,3,4,5]. In the rhesus macaque simian immunodeficiency virus (SIV) infection model and in persons living with HIV, early and persistent damage to the brain and other tissues are observed [2,3,6,7]. Damage to gut-associated lymphoid tissue results in acute and chronic immune activation, inflammation, and oxidative stress. These are linked to increased microbial translocation and associated end-organ complications, particularly in the CNS [1,2,8,9]. The maximum prevention of such complications likely requires protective therapies that are initiated concurrently with cART that suppress these pathologic pathways.

One approach to reducing oxidative-stress-associated injury is the activation of the host endogenous antioxidant response, which is mediated in part through the activation of the nuclear-factor-E2-related factor 2 (Nrf2)/antioxidant response element (ARE) pathway. In various animal models, activation of the ARE in the brain is associated with reduced neurodegeneration, whereas suppression of the ARE may promote neurodegeneration [10]. Activation of the Nrf2/ARE pathway to regulate oxidative stress and inflammation in the brain may promote axonal regeneration and block neurotoxicity [11,12,13,14,15].

The fumaric acid ester family, as represented by dimethyl fumarate (DMF) and monomethyl fumarate (MMF), which is the active in vivo metabolite of DMF, can activate Nrf2/ARE signaling, as well as non-Nrf2/ARE pathways, regulating the expression of antioxidant, anti-inflammatory, and cytoprotective genes [16,17,18,19]. DMF and MMF induce the expression of Nrf2/ARE-driven genes (NAD(p)H quinone dehydrogenase 1 (NQO1), glutathione peroxidase 1 (GPX1), peroxiredoxin 1 (RDX1), and heme oxygenase-1 (HO-1), among others) when applied to cultured cells or when administered to experimental animals [12,18,20]. DMF and MMF can also reduce the production of nitric oxide synthase and proinflammatory cytokines IL-1β, TNFα, and IL-6, and reduce NF-κB-mediated proinflammatory signaling [21,22,23,24]. Furthermore, DMF and MMF can suppress vascular cell adhesion molecule 1 (VCAM-1) expression in endothelial cells in vitro [25,26]. In the experimental autoimmune encephalitis (EAE) mouse model, the administration of DMF reduces macrophage infiltration into areas of demyelination [27,28], and in the LPS-induced neuroinflammation mouse model, DMF can reduce the expression of the microglial activation marker, Iba1 [29]. Based upon many such observations and human clinical therapeutic trials, oral DMF preparations have been approved for the treatment of multiple sclerosis [30,31]. The multiple immune-modulating effects of DMF have prompted investigations into its repurposing for use in other diseases that are associated with inflammation and oxidative stress, including HIV infection [32,33,34,35].

We previously identified the neuroprotective mechanistic effects of both DMF and MMF against HIV in our cell co-culture model containing both HIV-infected human macrophages and primary rodent neurons [20,36]. The HIV infection of macrophages results in a marked reduction in the expression of HO-1, which is associated with the release of neurotoxic levels of glutamate [20,36]. The exposure of these infected macrophages to DMF or MMF induces the expression of HO-1 and other ARE-driven enzymes and markedly reduces macrophage glutamate release, thus dramatically reducing excitotoxic neuronal injury. The selective induction of HO-1 expression in HIV-infected macrophages through targeted siRNA derepression confirms these HO-1 effects on glutamate release [36].

In this study, we determined whether DMF would induce antioxidant and anti-inflammatory responses within the brains of rhesus macaques during SIV infection, which is the non-human primate model of HIV infection. Studies in various rodent models of neurodegeneration and neuroinflammation have shown that DMF administration results in antioxidative and anti-inflammatory effects in the rodent brain (reviewed in [37]), with excellent tissue penetration [38]. We used the rhesus macaque CD8^+^ T lymphocyte depletion model of pathogenic SIV infection, as this model shows consistent brain inflammation and oxidative stress associated with a relatively rapid progression (weeks) to severe SIV-induced immune deficiency [39]. We reasoned that this accelerated pathogenesis model would be better suited for assessing the CNS effects of DMF treatment than an SIV infection model without CD8^+^ T lymphocyte depletion, which is associated with less consistent brain inflammation and oxidative stress. Such consistent neuropathogenic responses are particularly important in studies that are limited to the relatively small numbers of macaques that can be resourced for therapeutic studies [40].

## 2. Materials and Methods

### 2.1. Ethical Statement for Use of Nonhuman Primates

This study was performed following the Institutional Animal Care and Use Committee (IACUC) of Tulane University approved protocol, number A3180-01, in accordance with the Animal Welfare Act and other federal statutes and regulations relating to animals. The animals were housed at the Tulane National Primate Research Center (TNPRC) at Tulane University (Covington, LA, USA) following the guidelines established by the National Institutes of Health (NIH) and under the supervision of the Association for the Assessment and Accreditation of Laboratory Animal Care (AAALAC)-accredited Division of Animal Resources.

### 2.2. Administration of DMF to Rhesus Macaques

Based upon the approved DMF daily dose of 480 mg (≈7 mg/kg) in humans, we synthesized gelatin-coated capsules (30 mg DMF [Sigma, St. Louis, MO, USA]/methyl cellulose matrix) for equivalent dosing in macaques. The capsules were prepared by the Investigational Drug Service at the University of Pennsylvania. Crystalline DMF was mixed with methylcellulose, pressed into 30 mg capsules, and coated with gelatin (BSE-free, certified, pharmaceutical grade). To determine the DMF uptake in uninfected control macaques, DMF (30 mg) embedded into FLAVORx medication flavoring (Patterson veterinary, Greeley, CO, USA) was administered by gastric tube or by oral ingestion (one macaque each). For comparison, one macaque received a DMF capsule administered via a gastric tube. We used a 30 mg dose to analyze the single-dose kinetics of DMF absorption and metabolism to determine the lower expected peak plasma concentration of DMF dosing used in the study.

### 2.3. Detection and Kinetics of MMF in Rhesus Macaque Plasma

Blood was harvested at 0, 60, 120, 180, 240, 300, 360, and 480 min after the DMF administration. Plasma was assayed for MMF (Sigma, Inc.) via liquid chromatography–mass spectrometry (LC–MS, Agilent 6490 Triple Quad LC-MS/MS; Agilent Technologies, Santa Clara, CA, USA) using a labeled MMF standard curve. The concentration of MMF was determined using LC–MS through the Childrens Hospital of Philadelphia Metabolics Core [41]. An eight-point standard curve (concentrations from 10,000 to 78 ng/mL) was prepared by reconstituting MMF in macaque plasma, which was previously separated from untreated rhesus macaque blood, and detected using monomethyl fumarate-^13^C-D3. Plasma from DMF-treated animals was separated and samples were extracted with acetonitrile, dried, and reconstituted in acetonitrile solution. Each sample was then assayed using LC–MS to establish the MMF content.

### 2.4. DMF Treatment and SIV Infection

**Figure 1:**
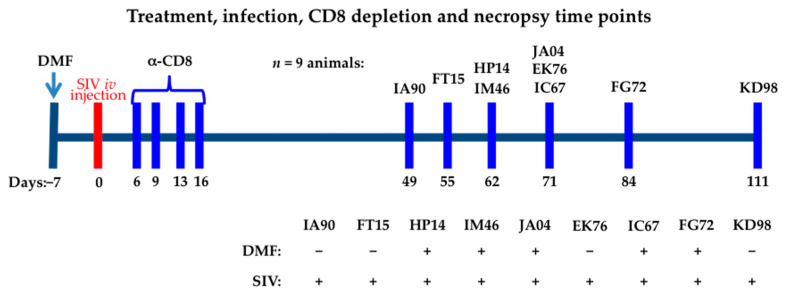
Schedule for dimethyl fumarate (DMF) treatment, anti-CD8 antibody treatments, simian immunodeficiency virus (SIV) inoculation, and necropsies of rhesus macaques. DMF treatment (90 mg total DMF daily, 5 animals DMF-treated, 4 untreated) was initiated seven days prior to the SIV infection, which was considered to be day “0.” On day “0” all animals (9 rhesus macaques) were intravenously infected with 100 TCID_50_ (50% tissue culture infectious dose) SIV_mac251_. CD8^+^ T lymphocyte depletion was achieved via treatment with anti-CD8 antibody infusions from 6 to 16 days post infection (dpi) via subcutaneous administration (10 mg/kg for the first dose and then subsequent doses at 5 mg/kg. Necropsies were performed at the indicated dpi thereafter. Individual animals are identified by unique letter–number combinations.

Nine healthy, Indian rhesus macaques (from 5 to 17 years old) were used. Animals were divided into two groups: (i) SIV+ and (ii) SIV+/DMF treated (Figure 1). Enteral DMF was administered twice daily, 60 mg in the morning and 30 mg in the evening (daily treatment of 90 mg, ≈7 mg/kg of body weight), starting 7 days prior to the SIV_mac251_ intravenous (iv) inoculation (100 TCID_50_ (50% tissue culture infectious dose] in 1 mL of sterile phosphate-buffered saline). All macaques selected were male, as sex is a known biological variable in Nrf2-regulated genes [42] and anti-oxidant responses in males and females [43]. The availability of rhesus macaques at the Tulane National Primate Research Center allowed all male animals to be selected for this study to eliminate confounding sex effects on the targeted antioxidant responses.

Animals were treated with the CD8^+^-T-lymphocyte-depleting antibody cM-T807 (provided by the Tulane Nonhuman Primate Reagent Resource Center) via subcutaneous injection, beginning at 6 days post infection (dpi) at a dose of 10 mg/kg. Subsequent doses (5 mg/kg) were applied at 9, 13, and 16 dpi.

### 2.5. Plasma and the Cerebrospinal Fluid (CSF) Viral Load

Animals were infected with SIV_mac251_ via iv infusion (100 TCID_50_) [44]. The plasma and CSF samples were collected at time points up until the day of necropsy (Figure 1). The plasma and CSF were immediately frozen (80 °C). The plasma and CSF viral loads were determined using the Quantitative Molecular Diagnostics Core AIDS and Cancer Virus Program at the Frederick National Laboratory for Cancer Research (Leidos Biomedical Research, Frederick, MD, USA). The presence of SIV RNA was determined with a sensitive (15 copies Eq/mL threshold sensitivity), quantitative real-time polymerase chain reaction (qPCR) assay that targets a region in SIV *gag* using primers and a TaqMan^®^ probe (ThermoFisher Scientific, Waltham, MA, USA). The following primer pairs were used: forward primer (SGAG21) 5′-GTCTGCGTCATPTGGTGCATTC-3′ and reverse primer (SGAG22) 5′-CACTAGKTGTCTCTGCACTATPTGTTTTG-3′, along with probe (pSGAG23) 5′-(FAM) CTTCPTCAGTKTGTTTCACTTTCTCTTCTGCG-(BHQ™1)-3 [45].

### 2.6. Brain and Peripheral Tissue Harvesting

Eleven brain regions were harvested at the necropsy time points (frontal cortex, third ventricle, thalamus, parietal cortex, subcortical white matter, cerebellum, brainstem, basal ganglia, caudate nuclei, temporal cortex, and occipital cortex). Thymus, liver, and spleen tissues were also collected. All tissues for Western blot or RNA analysis were immediately frozen (80 °C). Tissues for immunohistochemistry (IHC) staining and optical redox imaging were preserved in paraformaldehyde for paraffin embedding.

### 2.7. Western Blotting

Tissue lysates were prepared using homogenization (≈100 mg of tissue) via silica bead beating and sonication in a seven-volumes buffer (weight/volume) (10 mM Tris-HCl pH 7.8, 0.5 mM dithiothreitol, 5 mM MgCl_2_, 0.03% Triton X-100) containing a phosphatase inhibitor cocktail (PhosSTOP, Roche Diagnostics GmbH, Mannheim, Germany) and a protease inhibitor cocktail (cOmplete, Roche Diagnostics GmbH). The protein was quantified using the DC™ (detergent compatible) protein assay (Bio-Rad Laboratories, Hercules, CA, USA). Equivalent amounts of proteins were added to a Laemmli sample buffer (Sigma, Inc.) with 2.5% 2-mercaptoethanol (Bio-Rad laboratories) and then denatured at 95 °C for 10 min. Proteins were resolved on an SDS-PAGE gel and transferred overnight at 4 °C to poly(vinylidene fluoride) (PVDF) membranes (Merck Millipore Ltd., Carrigtwohill, Co. Cork, Ireland). The membranes were blocked with an Odyssey Blocking Buffer (PBS) (LI-COR Biosciences, Lincoln, NE, USA) and incubated with primary antibody overnight (4 °C) (Table 1 for the list of antibodies). TRDye-conjugated secondary antibodies (LI-COR Biosciences) were used to detect the primary antibody. Background-corrected signal quantification of protein bands was determined using Image Studio Lite software (LI-COR Biosciences). One sample prepared from mixing equal volumes of all samples (Mix) was used as a running and transfer control in all membranes. Each blot was normalized to that sample in each membrane, allowing for comparisons between all brain regions and animals.

### 2.8. Immunohistochemistry Analyses

One of two adjacent portions of each brain region per animal was fixed via immersion in 4% paraformaldehyde for 48 h, washed in PBS, and transferred to 70% ethanol before paraffin embedding and sectioning (8 μm). The remaining portion was frozen and processed for protein quantification, as described above. For the IHC staining, four sections from each region were harvested from each animal for labeling with each antibody. Sections (8 μm) were deparaffinized in xylene and rehydrated in ethanol and distilled water, quenched with 3% H_2_O_2_ for 30 min, and microwaved for 15 min in a 10 mM citrate buffer (pH 6) (Vector Laboratories, Inc., Burlingame, CA, USA) for epitope exposure. Primary antibodies (Table 1) and isotype-matched control antibodies were used at identical concentrations for staining with an overnight incubation (4 °C), two successive washes, and incubation with an appropriate biotin-conjugate secondary antibody. The signal was amplified using an avidin-biotin horseradish peroxidase system following the manufacturer’s instructions (Vectastain ABC kit, Vector Laboratories, Inc.). A chromophore reaction was developed with diaminobenzidine and hydrogen peroxide. All slides from both groups of animals (untreated vs. treated) and for each marker were stained at the same time and with the same batch of reagents. The sections were dehydrated and mounted with Cytoseal 60 (ThermoFisher). Images from all sections were taken using a digital slide scanner (Aperio AT2, Leica Biosystem, Wetzlar, Germany) at 20x magnification and were analyzed with QuPath software (version 0.2.0-m5, University of Edinburgh, Edinburgh, United Kingdom), as previously reported [46].

### 2.9. Optical Redox Imaging

The autofluorescence of protein-bound NADH and oxidized flavoproteins containing flavin adenine dinucleotide (Fp) of formalin-fixed-paraffin-embedded (FFPE) tissue slides (8 µm) was imaged and quantified, as previously described [47]. NADH signals were collected using bandpass filters with excitation (Ex) at 370–400 nm, and emission (Em) at 414–450 nm. The Fp signals were acquired with Ex: 450–488 nm and Em: 500–530 nm bandpass filters using a Zeiss wide-field microscope (Axio Observer.Z1/7, One North Broadway, White Plains, NY, 10601, USA). Tile images (pixel size 1.172 × 1.172 µm^2^) were taken using a 5×/0.16 NA objective with shading correction on the fly, followed by photostitching. The acquired images were processed with a customized Matlab^®^ (The MathWorks, Inc., 1 Apple Hill Dr, Natick, MA 01760, USA) routine to generate the redox images where the redox ratio Fp/(NADH + Fp) image was generated pixel-by-pixel from the NADH and Fp images and to obtain the mean value of each of the redox indices (NADH, Fp, the redox ratio) using global averaging [47,48,49]. Obvious fluorescent artifacts (in either channel) were removed during the imaging analysis.

### 2.10. Plasma and CSF Neurofilament Assay

The plasma and CSF collected from each animal at its necropsy time point were used to quantify NFL using a Singleplex Assay (Meso Scale Discovery, Rockville, MD, USA), as described in the R-PLEX™ protocol and as previously reported [6].

### 2.11. Data Acquisition and Statistical Analyses

Statistical analyses were performed using GraphPad Prism software (San Diego, CA, USA). Data were analyzed using unpaired and paired *t*-tests.

## 3. Results

### 3.1. Enteral Administration of a Human-Equivalent Therapeutic DMF Dose in Rhesus Macaques Produced Therapeutic Plasma Levels of MMF

**Figure 2:**
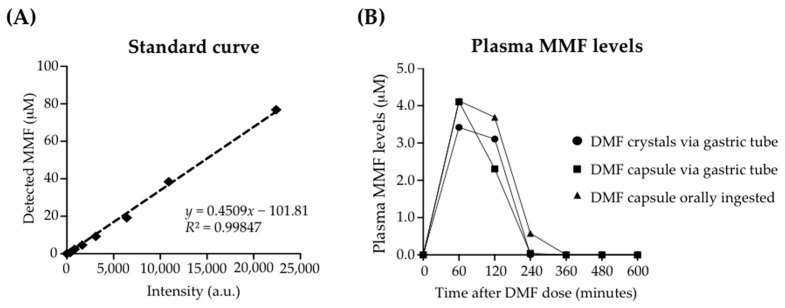
Plasma monomethyl fumarate (MMF) levels in rhesus macaques after a single enteral dose of DMF (30 mg). (**A**) An eight-point standard curve was prepared using serial dilutions (MMF concentrations from 77 to 0.6 μM). MMF was reconstituted in macaque plasma, which was previously separated from untreated rhesus macaque blood, and detected using liquid chromatography–mass spectrometry. (**B**) Each of three adult male rhesus macaques (8.1 kg, 9.1 kg, 11.4 kg) received a single 30 mg dose of DMF admixed in methylcellulose, either as a gelatin-coated capsule or as a crystalline suspension. One animal received the DMF capsule via a gastric tube. One animal received 30 mg of DMF embedded in a foodstuff, which was orally ingested. The remaining animal received a suspension of DMF crystals via a gastric tube. Plasma was separated from whole blood drawn at each indicated time point after the DMF administration. Samples were extracted with acetonitrile, dried, and reconstituted in acetonitrile solution. Each sample was assayed using LC–MS to establish the MMF content. Standard curves were prepared using monomethyl fumarate-^13^C-D3 for calibration.

Previous studies have confirmed the detection of MMF, and not DMF, in the plasma, serum, and whole blood of DMF-treated individuals [50,51,52]. Such a conversion of DMF to MMF occurs very rapidly (minutes) through hydrolysis-mediated intestinal and serum hydrolases [52]. To confirm the absorption of DMF in rhesus macaques, we administered a single DMF dose (30 mg) in each of three ways: (i) gastric tube delivery of a gelatin-coated capsule (DMF admixed with methylcellulose), (ii) oral ingestion of DMF mixed in a foodstuff, and (iii) gastric tube delivery of a DMF suspension (Figure 2). DMF treatments were continued up to the day of elective euthanasia and necropsy. Using LC–MS analysis, we detected plasma MMF at concentrations up to 4.1 μM at 60 min with each method of administration, followed by reduced plasma levels at 120 and 240 min. As previously reported, only MMF, and not DMF (not shown), was detected. This plasma MMF level was within the therapeutic plasma level range in humans treated for multiple sclerosis with oral DMF preparations [50,51]. We, therefore, implemented a treatment regimen of twice-daily DMF administration (similar to human regimens) of gelatin-encapsulated DMF (30 mg, 60 mg).

### 3.2. Plasma and Cerebrospinal Fluid SIV Levels Were Not Altered by the DMF Treatment

**Figure 3:**
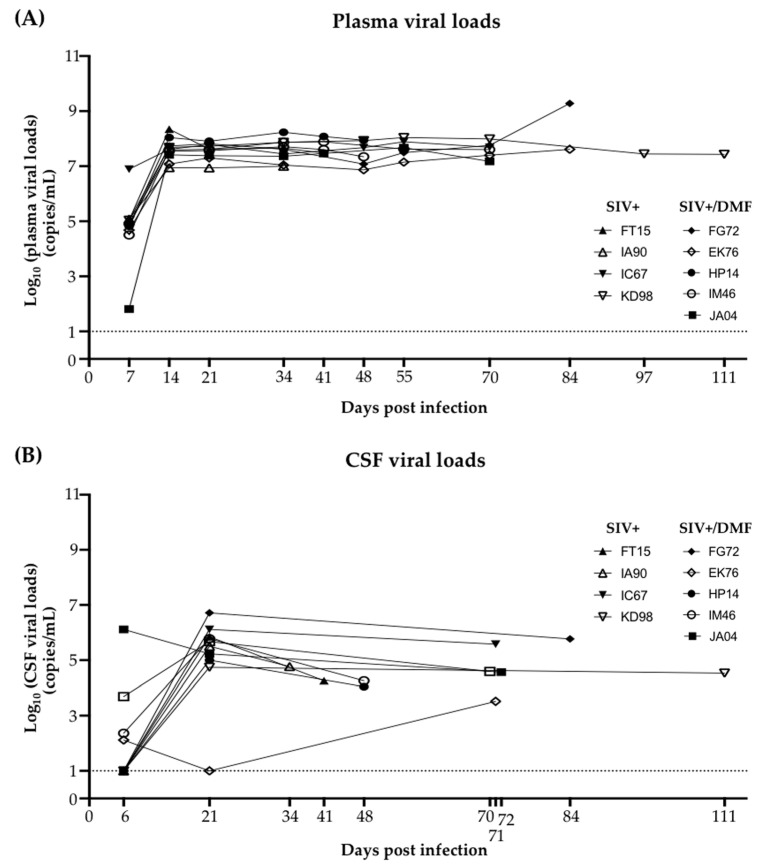
SIV RNA was detectable in plasma from all animals after 7 dpi and in cerebrospinal fluid (CSF) at the necropsy time points. (**A**) SIV plasma viral loads (RNA copies/mL) from each animal. SIV RNA was detectable from 7 dpi until the necropsy day. (**B**) The SIV CSF viral loads (RNA copies/mL) from each animal. SIV RNA was detectable in one animal at 6 dpi. One animal did not have detectable CSF SIV RNA until the necropsy day. Nine rhesus macaques, all SIV-infected, were used in the study (4 untreated animals and 5 DMF-treated (90 mg total DMF daily)). Each dot represents the value for each animal at each time point. Individual animals are listed by unique letter–number combinations.

The DMF treatment was initiated one week prior to the SIV infection, followed by CD8^+^ T lymphocyte depletion (Figure 1) and monitoring of the plasma and CSF viral loads (Figure 3). Plasma SIV was detected at 6 dpi in all animals (Figure 3A), while CSF SIV was detected in one animal at 6 dpi and all but one animal at 21 dpi (Figure 3B). At the necropsy time points, CSF SIV was detected in all animals. Over the course of the infection, the plasma SIV levels were approximately 1 log higher than those in CSF, as has previously been reported [53]. There was no significant difference between the SIV levels in DMF-treated vs. non-treated animals, which is consistent with our prior data showing no effect of DMF or MMF on HIV replication in HIV-infected macrophages [4].

### 3.3. DMF Treatment Induced Nrf2/ARE-Driven Gene Expression Throughout the Brain

**Figure 4:**
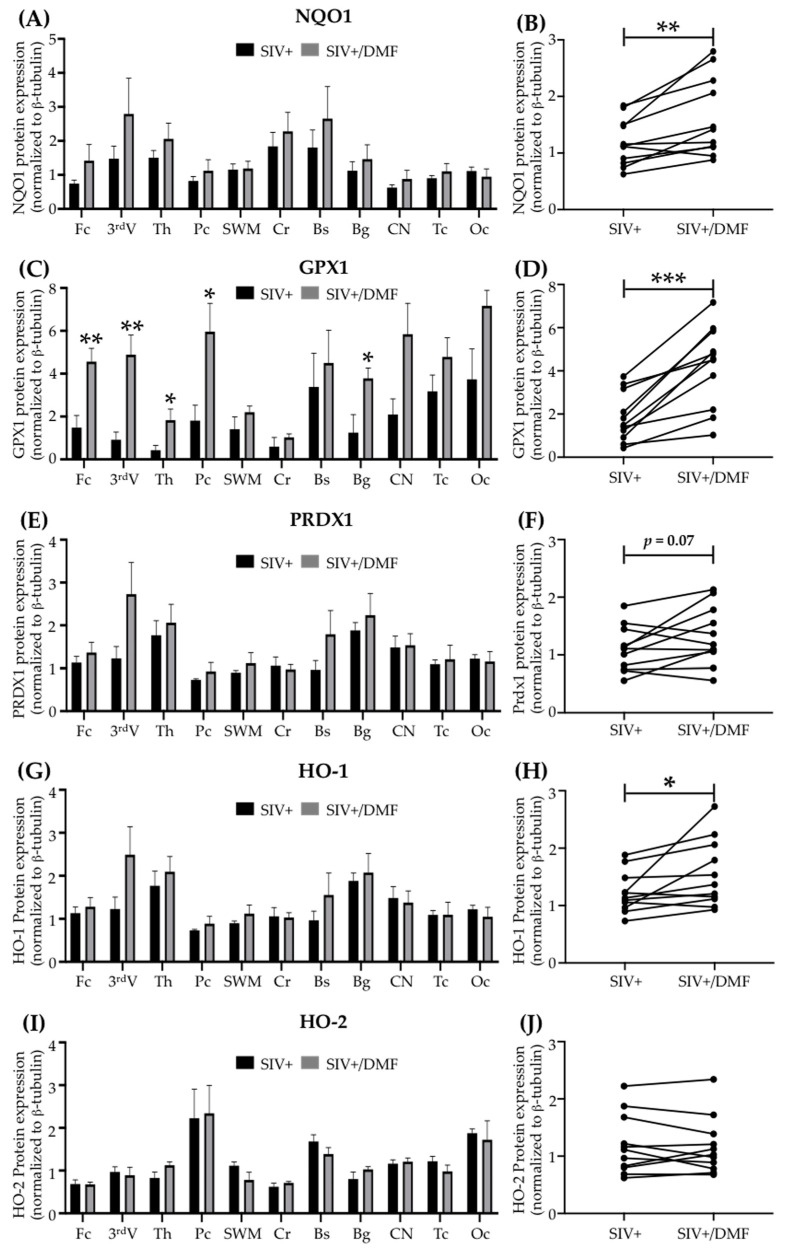
DMF treatment was associated with higher antioxidant enzyme expression in the brains of SIV-infected macaques. (**A**) No significant difference in NQO1 expression was observed in the individual regions using Student’s unpaired *t*-test (Fc: frontal cortex, 3^rd^V: third ventriculi, Th: thalamus, Pc: parietal cortex, SWM: subcortical white matter, Cr: cerebellum, Bs: brainstem, Bg: basal ganglia, CN: caudate nuclei, Tc: temporal cortex, Oc: occipital cortex). (**B**) A significant increase in overall mean NQO1 expression was observed with DMF treatment (paired *t*-test). (**C**) A significant increase in GPX1 expression was observed in the Fc, 3^rd^V, Th, Pc, and Bg with DMF treatment. (**D**) A significant increase in overall mean GPX1 expression was observed with DMF treatment. (**E**) No significant difference in PRDX1 expression was observed in individual regions. (**F**) A statistically non-significant (*p* = 0.07) increase in the overall mean PRDX1 expression was observed with DMF treatment. (**G**) No significant difference in HO-1 expression was observed in individual regions. (**H**) A significant increase in overall mean HO-1 expression was observed with DMF treatment. (**I**,**J**) No significant difference in HO-2 expression was observed in individual regions or overall in the brains with DMF treatment. Differences in expression within individual brain regions between both groups (DMF-treated and untreated) were evaluated using Student’s unpaired *t*-test and comparison between the mean of the expression for each enzyme in each group was done using Student’s paired *t*-test (* *p* < 0.05, ** *p* < 0.01, *** *p* < 0.001). Nine SIV-infected rhesus macaques (4 untreated animals and 5 DMF-treated (90 mg total DMF daily)). All quantifications are expressed as mean ± standard error of the mean (SEM). In the right panels, each dot represents the average for each region and group.

**Figure 5:**
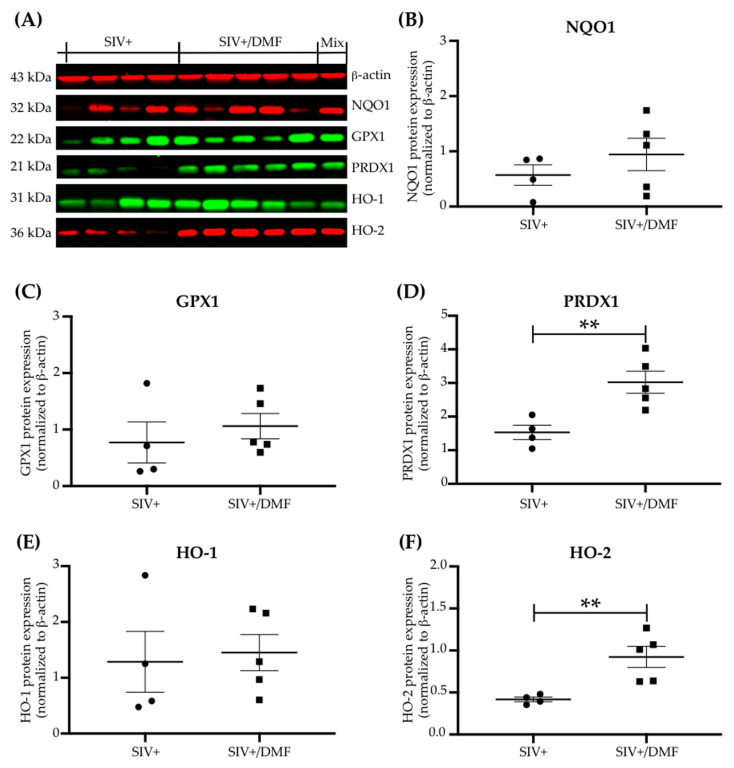
DMF treatment was associated with higher antioxidant enzyme expression in the spleens of SIV-infected macaques. (**A**) Representative immunoblot for the antioxidant enzymes assessed. β-Actin was used as a loading control. (**B**,**C**) No significant differences in the expression of NQO1 and GPX1, respectively, were observed in the spleens with DMF treatment. (**D**) A significant increase in the PRDX1 expression was observed with DMF treatment. (**E**) No significant difference in the expression of HO-1 was observed with DMF treatment. (**F**) A significant increase in the HO-2 expression was observed with DMF treatment. Spleen samples from nine SIV-infected rhesus macaques (4 untreated animals and 5 DMF-treated (90 mg total DMF daily)) were analyzed using Western blotting. Each dot represents the quantified value per animal. Statistical analyses were done using Student’s unpaired *t*-test, ** *p* < 0.01. All values are expressed as mean ± SEM.

DMF targets the Nrf2/ARE pathway, which involves multiple genes that protect cells from oxidative stress and injury [37,54,55,56,57,58,59], including antioxidant enzymes, such as NQO1 [60], GPX1 [61], PRDX1 [62], and HO-1 [63]. We quantified the antioxidant enzyme expression in eleven brain regions (Figure 4), as well as the spleen (Figure 5), thymus, and liver (Figure S1), and observed higher NQO1 (*p* < 0.01), GPX1 (*p* < 0.001), and HO-1 (*p* < 0.05) expression in the brains of DMF-treated animals compared to untreated animals (via comparing the means of expression from each region in each group using a paired *t*-test, Figure 4B,D,F,H,J). Individual regional changes associated with DMF treatment were observed only for GPX1 expression in the frontal cortex, third ventricle, thalamus, parietal cortex, basal ganglia, and caudate nucleus (Figure 4C,D). HO-2 expression was unchanged with DMF treatment in all brain regions analyzed (Figure 4I,J). Unlike the DMF treatment effects in the brain, the DMF treatment effects in the spleen were associated with higher expressions of PRDX1 and HO-2 and no changes in NQO1, GPX1, or HO-1 (Figure 5A–F, unpaired *t*-test, *p* < 0.01). No changes in any of these enzymes were observed in the liver or thymus (Figure S1).

### 3.4. DMF Treatment Was Associated with a Lower Expression of DNA and Protein Oxidation Markers and a More Reduced Brain Redox State

**Figure 6:**
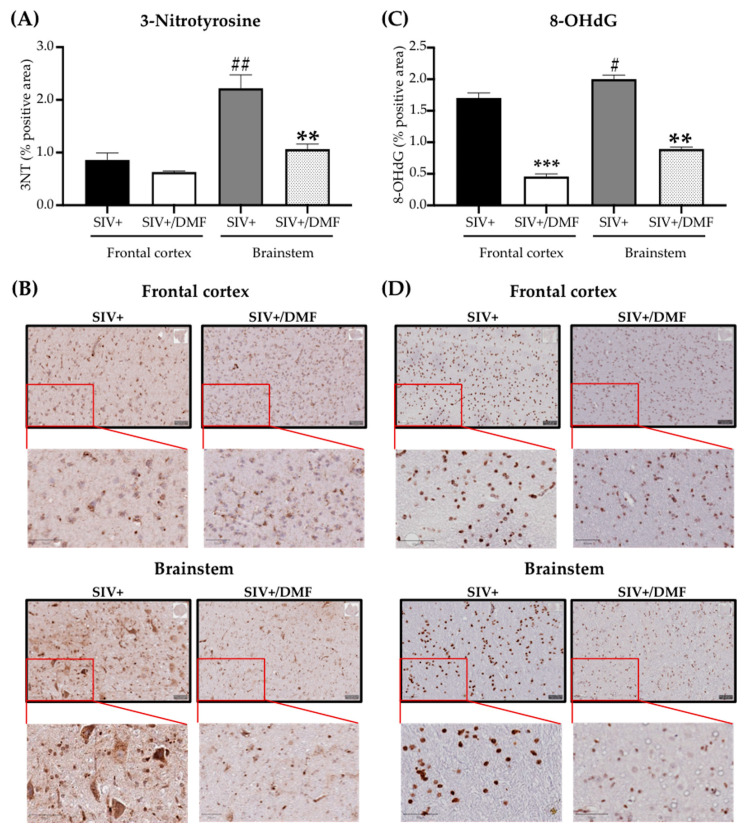
DMF treatment was associated with the reduced expression of markers of DNA and protein oxidation in the frontal cortexes and brainstems of SIV-infected macaques. (**A**) Quantification of 3NT (oxidized proteins) in the frontal cortex (left) and brainstem (right) showed reduced expression in the brainstem with DMF treatment. The 3NT labeling was significantly higher in the brainstem compared to the frontal cortex in SIV infection (statistical analysis was done with Student’s unpaired *t*-test, ** *p* < 0.01 comparing SIV+ vs. SVI+/DMF in the brainstem; ^##^ *p* < 0.01 comparing the frontal cortex vs. brainstem in the SIV+ group). (**B**) Representative labeling for 3NT in the frontal cortex (top panels) and brainstem (bottom panels). (**C**) Quantification of 8-OHdG (oxidized DNA) in the frontal cortex (left) and brainstem (right) showed reduced expression in the frontal cortex and brainstem with DMF treatment. 8-OHdG labeling was significantly higher in the brainstem compared to the frontal cortex in SIV infection (statistical analysis was done with Student’s unpaired *t*-test, ** *p* < 0.01 and *** *p* < 0.001 comparing SIV+ vs. SVI+/DMF; ^#^ *p* < 0.05 comparing the frontal cortex to the brainstem in the SIV+ group). (**D**) Representative labeling for 8-OHdG in the frontal cortex (top panels) and brainstem (bottom panels). Four sections from each region and animal were used to be labeled with each antibody. Nine SIV-infected rhesus macaques were used in the study (4 untreated animals and 5 DMF-treated (90 mg total DMF daily)). Images from all sections were taken using a digital slide scanner (Aperio AT2, Leica Biosystem, Wetzlar, Germany) at 20x magnification and were analyzed with QuPath software (version 0.2.0-m5), as previously reported [46]. Quantifications are expressed as the mean percentage of positive staining from all the stained areas ± SEM.

**Figure 7:**
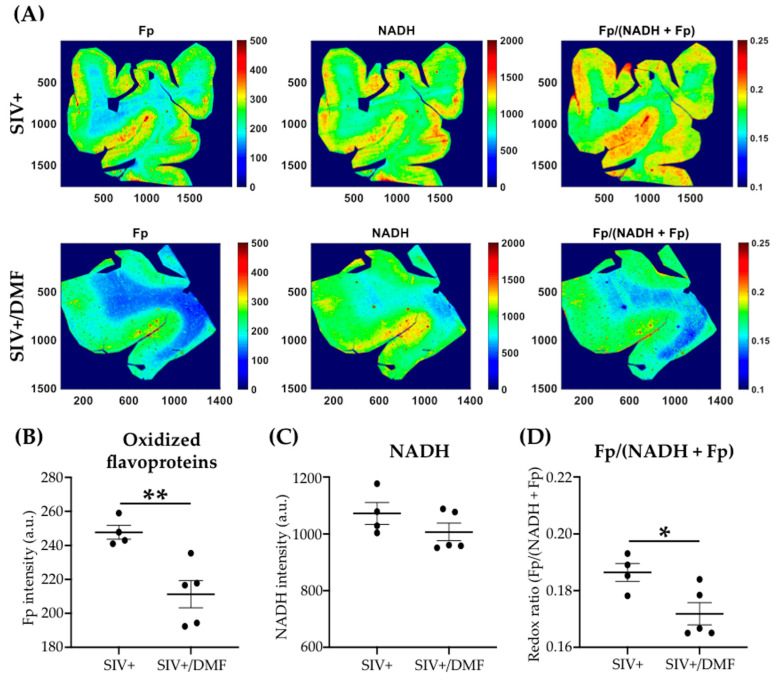
DMF treatment was associated with lower oxidized flavoprotein (Fp) levels and redox ratio Fp/(NADH + Fp) in the frontal cortex of SIV-infected macaques. (**A**) Representative images (frontal cortex) of autofluorescence of Fp (left), NADH (center) and Fp/(NADH + Fp) (right). The “*x*” and ”*y*” coordinates of the image matrices are shown. Numbers and color bars on the right of each image indicate the range of the index being displayed. (**B**) Quantification of the Fp autofluorescence showed lower Fp levels with DMF treatment (statistical analysis was done with Student’s unpaired *t*-test, ** *p* < 0.01). (**C**) No significant difference in NADH levels was observed with DMF treatment. (**D**) Quantification of Fp/(NADH+Fp) showed a more reduced redox ratio being associated with DMF treatment, which was indicative of the oxidative stress level (statistical analysis was done using Student’s unpaired *t*-test, * *p* < 0.05). Nine SIV-infected rhesus macaques were used in the study (4 untreated animals and 5 DMF-treated (90 mg total DMF daily)). Intensities are in arbitrary units (a.u.) and all redox indices are expressed as the mean ± SEM.

**Figure 8:**
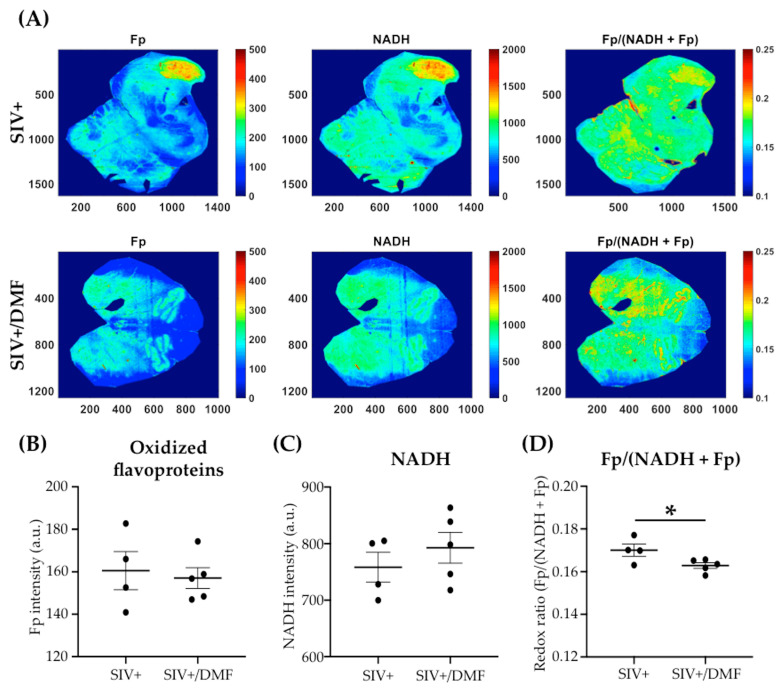
DMF treatment was associated with a lower redox ratio Fp/(NADH + Fp) in the brainstem of SIV-infected macaques. (**A**) Representative images (brainstem) of the autofluorescence of Fp (left), NADH (center), and Fp/(NADH + Fp) (right). The “*x*” and “*y*” coordinates of the image matrices are shown. Numbers and color bars on the right of each image indicate the range of the redox index being displayed. (**B**) Quantification of the Fp autofluorescence showed no significant change in Fp levels with DMF treatment (statistical analysis was done with Student’s unpaired *t*-test). (**C**) No significant difference in NADH levels was observed with DMF treatment. (**D**) Quantification of Fp/(NADH + Fp) showed that the DMF treatment was associated with a lower redox ratio, which was indicative of a lower oxidative stress level (statistical analysis was done with Student’s unpaired *t*-test, * *p* < 0.05). Nine SIV-infected rhesus macaques were used in the study (four untreated animals and five DMF-treated (90 mg total DMF daily)). Intensities are in arbitrary units (a.u.) and all redox indices are expressed as the mean ± SEM.

We applied a complementary approach of immunohistochemical labeling and optical redox imaging in sections from the frontal cortex and brainstem to determine the levels of oxidized proteins (3NT) and DNA (8-OHdG) (Figure 6) and the redox state (Figure 7; Figure 8). Notably, higher levels of protein (Figure 6A,B) and DNA (Figure 6C,D) oxidation were observed in the brainstem compared to the frontal cortex. Furthermore, DMF treatment was associated with lower protein oxidation in the brainstem (Figure 6A, *p* < 0.05) and lower DNA oxidation in both regions (frontal cortex *p* < 0.001 and brainstem *p* < 0.01) (Figure 6C,D).

To determine the effects of DMF treatment on the brain redox status, we quantified the autofluorescence of protein-bound NADH and Fp using the optical redox imaging of FFPE tissue slide technique [47]. NADH and Fp signals reflect the mitochondrial redox state and can detect the tissue redox contrast between different physiological/pathological states and therapeutic responses [64,65]. Within the frontal cortex, we observed lower Fp levels with the DMF treatment (Figure 7A,B, *p* < 0.01) and no change in NADH levels (Figure 7A,C). The redox ratio Fp/(NADH + Fp) was significantly lower in the DMF-treated group (Figure 7D, *p* < 0.05). DMF treatment did not induce significant changes in Fp or NADH levels within the brainstem (Figure 8A–C), but it was associated with a significantly lower redox ratio, albeit with a small effect size (Figure 8D, *p* < 0.05).

### 3.5. No Toxic Effect Was Associated with DMF Treatment

**Figure 9:**
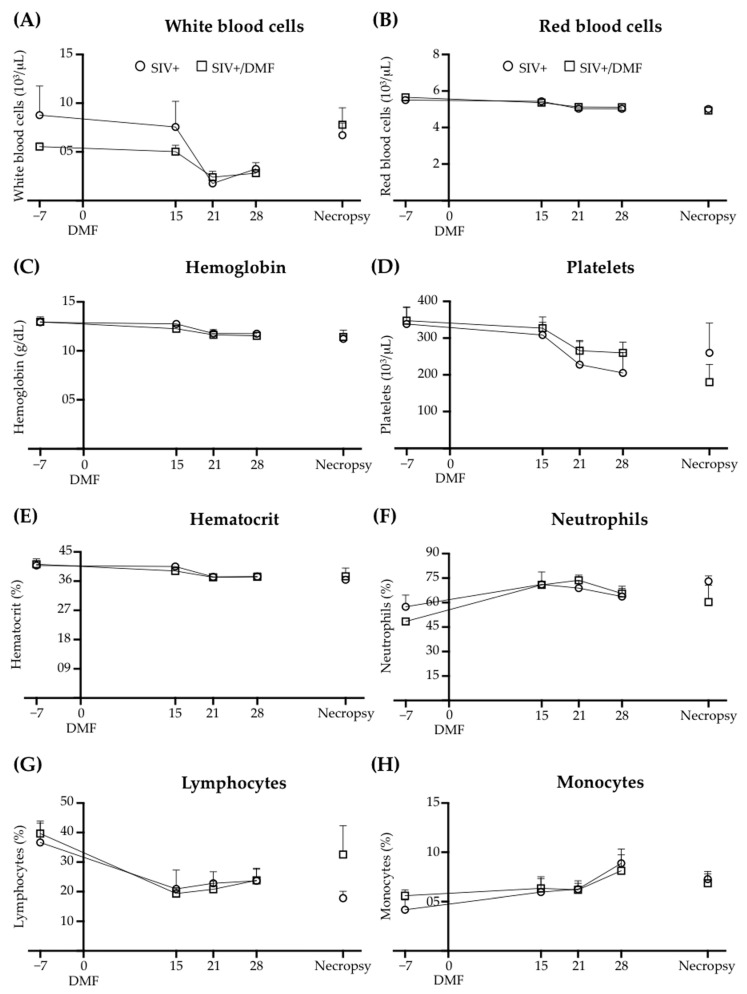
DMF treatment did not associate with changes in blood cell counts. (**A**) White blood cell count. (**B**) Red blood cell count. (**C**) Hemoglobin level. (**D**) Platelet count. (**E**) Hematocrit level in total blood. (**F**) Percent of neutrophils from total white cells. (**G**) Percent of lymphocytes from total white cells. (**H**) Percent of monocytes from total white cells. Blood parameters were assessed at time points (days) from the day of initiation of the DMF treatment. Each point represents the average for each treatment group at that time point. Nine SIV-infected rhesus macaques were used in the study (4 untreated animals and 5 DMF-treated (90 mg total DMF daily)). All values are expressed as mean ± SEM.

**Figure 10:**
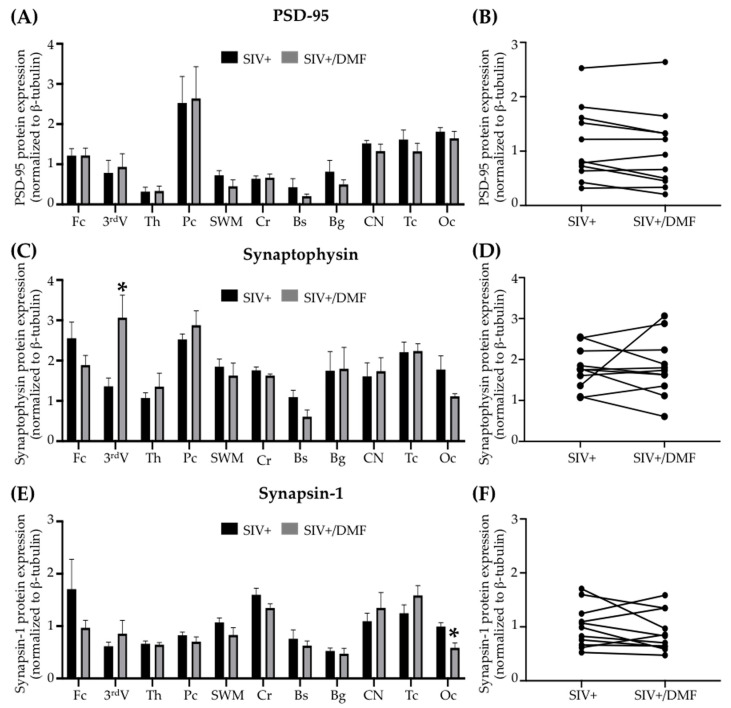
DMF treatment was associated with limited regional changes in expression of the synaptic proteins synaptophysin and synapsin I, but not PSD-95, in the brains of SIV-infected macaques. (**A,B**) No significant differences in PSD-95 (post-synaptic protein) expression were observed in individual regions or overall in the brains with DMF treatment. (**C**) A significant increase in synaptophysin (pre-synaptic protein) expression was observed in the third ventricle (Student’s unpaired *t*-test, * *p* < 0.05) but without a difference in the overall mean brain expression (**D**) with DMF treatment. (**E**) A significant decrease in synapsin 1 (pre-synaptic protein) expression was observed in the occipital cortex (Student’s unpaired *t*-test, * *p* < 0.05) but without a difference in the overall mean brain expression (**F**) with DMF treatment. Eleven brain regions from nine SIV-infected rhesus macaques (4 untreated animals and 5 DMF-treated (90 mg total DMF daily)) were analyzed using Western blotting. In the right panels, each dot represents the mean expression of one brain region from all animals in the group. Fc: frontal cortex, 3^rd^V: third ventricle, Th: thalamus, Pc: parietal cortex, SWM: subcortical white matter, Cr: cerebellum, Bs: brainstem, Bg: basal ganglia, CN: caudate nuclei, Tc: temporal cortex, Oc: occipital cortex. All values are expressed as mean ± SEM.

**Figure 11:**
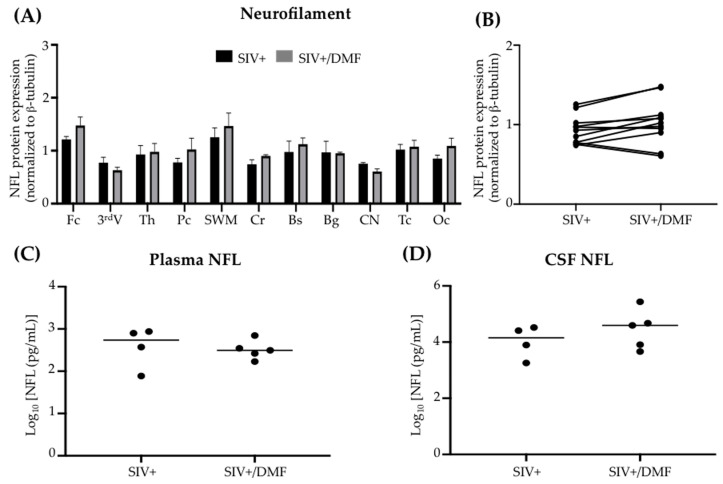
DMF treatment was not associated with changes in NFL. (**A**,**B**) NFL in the brain tissue, as determined using Western blot. Eleven brain regions from 9 SIV-infected rhesus macaques (4 untreated animals and 5 DMF-treated (90 mg total DMF daily)) were analyzed. In panel (**B**), each dot represents the mean average for each region and group. (**C**) The plasma and (**D**) cerebrospinal fluid (CSF) NFL did not change with the DMF treatment. NFL was quantified using a Singleplex Assay (Meso Scale Discovery, Rockville, MD, USA). Each dot represents the NFL value for each animal. Fc: frontal cortex, 3^rd^V: third ventricle, Th: thalamus, Pc: parietal cortex, SWM: subcortical white matter, Cr: cerebellum, Bs: brainstem, Bg: basal ganglia, CN: caudate nuclei, Tc: temporal cortex, Oc: occipital cortex. All values are expressed as mean ± SEM.

DMF treatment was not associated with changes in the peripheral blood hematologic counts (Figure 9), brain neuronal markers (synaptic proteins, Figure 10), or NFL in the brain tissue, plasma, or CSF (Figure 11). One DMF-treated SIV-infected animal developed a B cell lymphoma, commonly observed in immune-deficient SIV-infected macaques.

### 3.6. DMF Treatment Was Associated with a Higher Expression of VCAM-1 and ICAM-1

**Figure 12:**
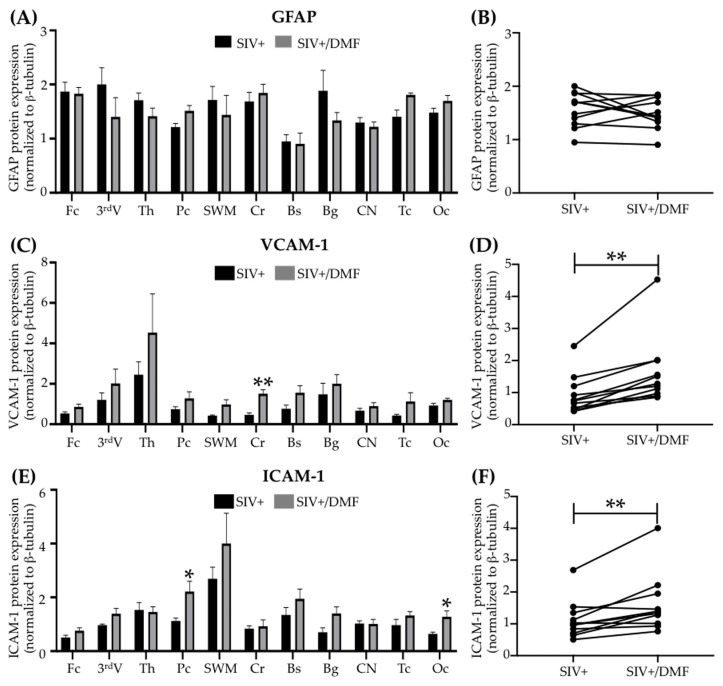
DMF treatment was associated with higher VCAM-1 and ICAM-1 expression in the brains of SIV-infected macaques. (**A**,**B**) No significant differences in the GFAP expression were observed in individual regions or overall in the brains with DMF treatment. (**C**) A significant increase in VCAM-1 expression in the cerebellum (Student’s unpaired *t*-test, ** *p* < 0.01) and in the overall mean expression (**D**) with DMF treatment (Student’s paired *t*-test, ** *p* < 0.01). (**E**) A significant increase in the ICAM-1 expression in the parietal and occipital cortexes (Student’s unpaired *t*-test, * *p* < 0.05) and in the overall mean expression (**F**) with DMF treatment (Student’s paired *t*-test, ** *p* < 0.01). Eleven brain regions from 9 SIV-infected rhesus macaques (4 untreated animals and 5 DMF-treated (90 mg total DMF daily)) were analyzed using Western blotting. In the right panels, each dot represents the mean expression of one brain region from all animals in the group. Fc: frontal cortex, 3^rd^V: third ventricle, Th: thalamus, Pc: parietal cortex, SWM: subcortical white matter, Cr: cerebellum, Bs: brainstem, Bg: basal ganglia, CN: caudate nuclei, Tc: temporal cortex, Oc: occipital cortex. All values are expressed as mean ± SEM.

**Figure 13:**
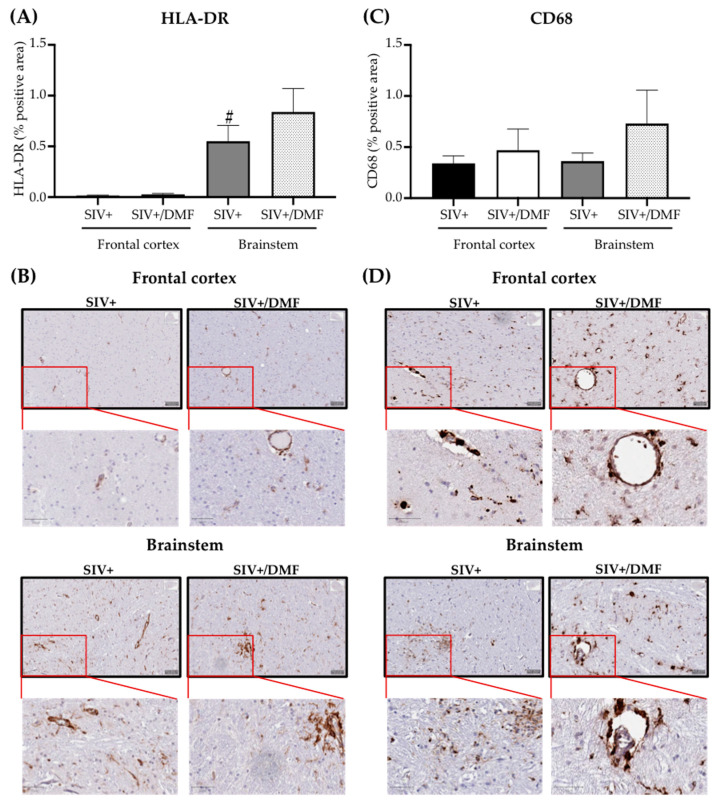
DMF treatment was not associated with the altered brain expression of HLA-DR (monocyte/microglia activation) or CD68 (monocyte marker). (**A**) Quantification of HLA-DR in the frontal cortex and brainstem. The brainstem showed a higher expression of HLA-DR than the frontal cortex (statistical analysis was done with Student’s unpaired *t*-test, ^#^ *p* < 0.05 comparing the frontal cortex vs. the brainstem in the SIV+ group) and the DMF treatment showed no effect on the HLA-DR expression. (**B**) Representative labeling for HLA-DR in the frontal cortex (top panels) and brainstem (bottom panels). (**C**) Quantification of CD68 in the frontal cortex and brainstem. The DMF treatment showed no effect on the CD68 expression. (**D**) Representative labeling for CD68 in the frontal cortex (top panels) and brainstem (bottom panels). Four contiguous sections from each region were harvested from each animal for labeling with each antibody. Nine SIV-infected rhesus macaques were used in the study (4 untreated animals and 5 DMF-treated (90 mg total DMF daily)). Images from all sections were taken using a digital slide scanner (Aperio AT2, Leica Biosystem, Wetzlar, Germany) at 20× magnification and were analyzed with QuPath software (version 0.2.0-m5), as previously reported [46]. Quantifications are expressed as the mean percent of positive staining from all the stained areas ± SEM.

DMF treatment was associated with no changes in the expression of GFAP (glial fibrillary acidic protein) and an increased expression of VCAM-1 (*p* < 0.01) and ICAM-1 (intercellular adhesion molecule 1, *p* < 0.01) (Figure 12). DMF treatment was not associated with changes in HLA-DR (human leukocyte antigen-DR isotype) or CD68 (Figure 13).

## 4. Discussion

The persistent risk for brain injury and associated neurocognitive impairment in persons living with HIV, despite the suppression of virus replication with antiretroviral therapy, underscores the need for the identification of adjunctive neuroprotective strategies [66,67]. Abundant evidence, including data from our studies, suggests that oxidative stress and neuroinflammation contribute to the risk for brain injury in acute and chronic HIV and SIV infections [4,6,20,68,69,70,71]. To address the need for identifying an effective adjunctive neuroprotection strategy, we examined the ability of DMF—an FDA-approved, anti-oxidative, and anti-inflammatory drug—to limit brain oxidative stress and neuroinflammation in chronically SIV-infected rhesus macaques, which is the non-human primate model of HIV infection.

To mimic the robust oxidative stress and neuroinflammation that is associated with HIV infection of the brain, we used the immune-deficient (CD8^+^ T lymphocyte depletion) model of SIV neuropathogenesis, which consistently produces the robust brain inflammation that is associated with SIV replication [39]. We approximated the DMF doses used in humans for the treatment of multiple sclerosis (≈7 mg/kg total daily dose) through enteral administration in the macaques and confirmed the single-dose kinetics of DMF absorption and metabolism, as indicated by the appearance of therapeutic levels of DMF’s biologically active in vivo metabolite, namely, MMF, in the plasma of uninfected animals prior to their entry into the infection arm of the study [72]. We detected the MMF plasma levels (≈4.1 μM) 60 min after the administration of DMF, followed by reduced levels at 120 and 240 min, which is consistent with prior studies in humans [50,51,52]. Numerous published studies, including our own, have demonstrated physiological antioxidant and anti-inflammatory responses in cultured cells that were exposed to MMF in this concentration range, thus suggesting that we can indeed achieve therapeutic levels in macaques when dosing with DMF concentrations used in humans [20]. Although we did not determine MMF levels in brain tissue or CSF, DMF treatments in animal models have been shown to achieve therapeutic levels in brain tissue [12,73]. Similar results have been shown by the nanoparticle-mediated oral delivery of formulated DMF in rodents detecting MMF in the brain [74]. Thus, our study contextualized the biological parameters of effective human therapeutic applications of DMF therapy for neuroprotection in the macaque model of HIV neuropathogenesis.

When comparing the DMF-treated vs. untreated SIV-infected animals, we observed the following associations with the DMF treatment: (i) higher brain expression of antioxidant enzymes NQO1, GPX1, and HO-1; (ii) lower brain expression of protein and DNA oxidation markers (3NT (proteins), 8-OHdG (DNA)); (iii) a more reduced redox state in both the frontal cortex and brainstem; (iv) higher spleen expression of antioxidant enzymes PRDX1 and HO-2; (v) no differences in the CSF and plasma NFL levels or SIV viral loads; (vi) no differences in the peripheral blood hematologic counts. Moreover, our analyses included 11 brain regions involving cortical and brainstem anatomical sites, which would identify global brain responses to DMF treatment. These observations suggest that chronic DMF administration at human-equivalent therapeutic doses to SIV-infected, immune-deficient rhesus macaques safely induced robust antioxidant responses throughout the brain.

We saw no evidence for neurotoxic effects, based upon the CSF and plasma NFL levels, although we cannot conclude that DMF demonstrated a protective effect against neuronal injury in these animals. We also found no evidence for anti-inflammatory effects from the DMF treatment in these animals. The lack of evidence in this study for significant neuroprotective or anti-inflammatory effects of DMF could be a consequence of the accelerated systemic and CNS disease progression in this SIV macaque model. SIV-infected macaques exhibit virological, immunological, and clinical manifestations that are similar to HIV-1 infected humans on a shorter time scale of 2 to 3 years. However, in CD8^+^-T-lymphocyte-depleted models, the SIV disease timeline is significantly accelerated (<6 months) with increased viremia, neuroinflammation, and CNS pathology, including SIV encephalitis [39]. This more fulminant disease model may obscure the anti-inflammatory and neuroprotective effects of DMF. Thus, whether DMF has the potential to prevent neuroinflammation and neuronal injury in SIV/HIV infection remains to be determined and future studies should examine DMF’s effects on neuroinflammation and brain injury in immune-competent macaques at early time points prior to significant disease progression.

Our demonstration of higher brain expression of antioxidant enzymes, lower DNA and protein oxidation, and a more reduced redox state in DMF-treated SIV-infected animals suggests a direct link between enzyme induction and reduced oxidative stress and injury. Among the enzymes induced were NQO1, GPX1, HO-1, and PRDX1. NQO1 is important in neuroprotection, particularly within dopaminergic regions, because of its ability to reduce reactive oxygen species (ROS), reduce/detoxify dopamine metabolites, and stabilize p53 [75]. GPX1 is a major detoxifier of peroxides, including lipid peroxides, and it is expressed in the nucleus, cytoplasm, and mitochondria; it is a sensitive and specific suppressor of ROS production and oxidative damage [61]. It is particularly important for mitochondrial H_2_O_2_ scavenging [76]. It has been shown that fumarate interacts directly with GPX1 to induce GPX1 activation [77]. Furthermore, DMF treatment has been shown to increase levels of reduced glutathione (GSH), which reduces peroxynitrite and the associated oxidative injury cellular injury [19]. HO-1 serves a critical function in heme detoxification (a major intra- and extracellular pro-oxidant), with a linkage to neuronal injury, recovery, and neuroprotection [78,79,80,81], especially in persons living with HIV [4,34,36,68,69]. PRDX1 is present in glial cells, primarily in the nucleus, cytosol, and mitochondria, and it removes peroxides, including H_2_O_2_, and peroxynitrite [82]. It is an effective suppressor of microglial activation [83,84] and it serves protective functions in neurodegenerative diseases [82]. Thus, there are numerous enzymatic pathways that may be activated by DMF treatment that could lead to a state of reduced oxidative stress in SIV infection, and presumably HIV infection.

We speculate that a major effect of DMF treatment is the reduction of mitochondrial oxidative injury. Particularly relevant to this are the nicotinamide adenine dinucleotide redox pair (NAD^+^/NADH) and the flavin adenine dinucleotide redox pair (FAD/FADH_2_). NADH and FADH_2_ are important electron donors in oxidative processes in the mitochondria [85,86]. Both redox pairs have critical roles in regulating the mitochondrial redox state and cellular metabolism [87,88,89,90], which generates ROS that can lead to increased oxidative stress when the redox homeostasis is dysregulated [91,92,93]. The optical redox ratio Fp/(NADH + Fp) linearly correlates with the NAD^+^/(NADH + NAD^+^) ratio that is determined using liquid chromatography–mass spectrometry and reflects the mitochondrial redox state [94,95]. A higher Fp or lower NADH or higher optical redox ratio corresponds to a higher mitochondrial ROS level; either oxidative or reductive extreme correlates with a higher ROS level [48,96] and a more oxidized redox state (a higher redox ratio) is associated with neurodegeneration [97,98].

## 5. Conclusions

Our results suggest a potential therapeutic benefit of DMF treatment in persons living with HIV, as oxidative stress in the periphery and the CNS are common features of HIV infection [4,20,36,68,70,71,99,100,101,102]. The DMF-treated macaques used in this study were severely immune-deficient (secondary to CD8^+^ T lymphocyte depletion) with high SIV loads, and DMF treatment showed no apparent adverse effects above those attributable to SIV infection. This suggests its potential safe use in persons living with HIV. Notably, in such groups of people who achieve virus suppression with cART and who subsequently undergo organ transplantation and associated immunosuppressive therapy, survival rates are equivalent to those of people without HIV [103]. We, therefore, have suggested the potential safe use of DMF in persons living with HIV as an adjunctive neuroprotective therapy [34]. Whether DMF prevents neuronal injury in SIV-infected macaques remains to be determined, where this is a future goal for testing DMF effects on early brain injury in SIV infection in immune-competent macaques as a model for neuroprotection in acute HIV infection [6].

## Figures and Tables

**Table 1:** Information regarding the antibodies used in the Western blotting and immunohistochemistry (IHC).

Antibody	Host	Isotype	Molecular Size	Dilution	Cat. No	Company
Anti-HO-1	Rabbit	Polyclonal	31 kDa	1:500	SPA-894	ENZO Life Sciences
Anti-HO-2	Mouse	Monoclonal IgG2a	36 kDa	1:1000	MA5-25749	ThermoFisher
Anti-PRDX1	Rabbit	Monoclonal	22 kDa	1:1000	8732	CST
Anti-NQO1	Mouse	Monoclonal IgG1	32 kDa	1:5000	ab28947	Abcam
Anti-GPX1	Rabbit	Monoclonal	22 kDa	1:2000	3286S	CST
Anti-GFAP	Mouse	Monoclonal IgG1	48 kDa	1:2000	3670S	CST
Anti-VCAM-1	Rabbit	Monoclonal	81 kDa	1:1000	ab134047	Abcam
Anti-ICAM-1	Rabbit	Monoclonal	58 kDa	1:1000	ab109361	Abcam
Anti-PSD-95	Mouse	Monoclonal IgG2a	95 kDa	1:1000	MAB1596	Sigma, Inc.
Anti-synaptophysin	Mouse	Monoclonal IgG1	37 kDa	1:1000	ab8049	Abcam
Anti-synapsin 1	Rabbit	Monoclonal	75 kDa	1:1000	5297S	CST
Anti-β-tubulin	Mouse	Monoclonal IgG2b	50 kDa	1:10000	86298S	CST
Anti-NFL	Rabbit	Monoclonal	66 kDa	1:10000	ab52989	Abcam
Anti-β-tubulin	Rabbit	Monoclonal	50 kDa	1:3000	2128S	CST
Anti-β-actin	Mouse	Monoclonal IgG1	42 kDa	1:30000	A4441	Sigma, Inc.
Anti-8-OHdG ^1^	Mouse	Monoclonal IgG1	N/A	1:100000	ab48508	Abcam
Anti-3NT ^1^	Mouse	Monoclonal IgG2a	N/A	1:2500	ab61392	Abcam
Anti-HLA-DR ^1^	Mouse	Monoclonal IgG2b	N/A	1:500	14-9956-82	ThermoFisher
Anti-CD68 ^1^	Mouse	Monoclonal IgG3 kappa	N/A	1:200	M087629-2	Agilent Technologies

ENZO Life Sciences (Farmingdale, NY, USA). Abcam (Cambridge, United Kingdom). CST: Cell Signaling Technologies (Danvers, MA, USA). N/A: not applicable; ^1^ used for IHC.

## Data Availability

The data presented in this study are available within the article and in supplementary material.

## Supplementary Materials

The following are available online at https://www.mdpi.com/2076-3921/10/3/416/s1, Figure S1: DMF treatment does not associate with changes in expression of antioxidant enzymes in liver or thymus.
