# Supplementary material for: Dimethyl Fumarate, an Approved Multiple Sclerosis Treatment, Reduces Brain Oxidative Stress in SIV-Infected Rhesus Macaques: Potential Therapeutic Repurposing for HIV Neuroprotection
Source: Antioxidants (Basel). 2021 Mar 9;10(3):416. doi: 10.3390/antiox10030416 (PMC7998206; doi:10.3390/antiox10030416)
Supplement: Supplementary file 1 [file antioxidants-10-00416-s001.pdf]

Supplementary Materials

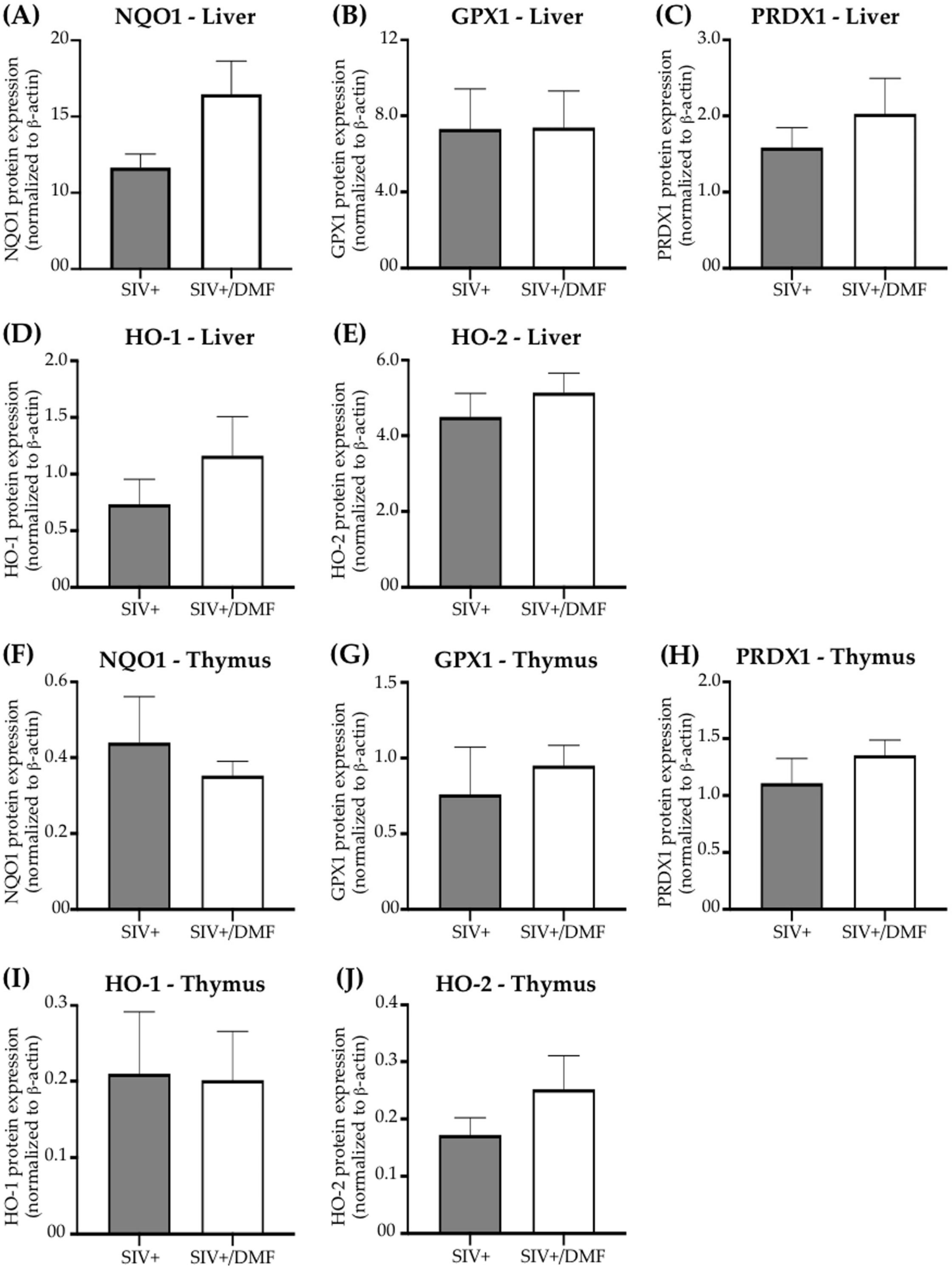

Figure S1. DMF treatment does not associate with changes in expression of antioxidant enzymes in liver **(A–E)** and thymus **(F–J)** in SIV-infected macaques. Nine SIV-infected rhesus macaques were used in this study (4 animals untreated and 5 DMF-treated [90 mg total daily dose]). All quantifications are expressed as mean ± SEM.
